# Correction: Candidate variants in DNA replication and repair genes in early-onset renal cell carcinoma patients referred for germline testing

**DOI:** 10.1186/s12864-023-09486-z

**Published:** 2023-07-10

**Authors:** Elena V. Demidova, Ilya G. Serebriiskii, Ramilia Vlasenkova, Simon Kelow, Mark D. Andrake, Tiffiney R. Hartman, Tatiana Kent, James Virtucio, Gail L. Rosen, Richard T. Pomerantz, Roland L. Dunbrack, Erica A. Golemis, Michael J. Hall, David Y. T. Chen, Mary B. Daly, Sanjeevani Arora

**Affiliations:** 1 Cancer Prevention and Control Program, Fox Chase Cancer Center, 333 Cottman Avenue, Philadelphia, PA 19111 USA; 2 Kazan Federal University, Kazan, 420008 Russia; 3 Program in Cancer Signaling and Microenvironment, Fox Chase Cancer Center, Philadelphia, PA 19111 USA; 4 Department of Biochemistry and Molecular Biophysics, University of Pennsylvania, Philadelphia, PA 19104 USA; 5 Arcadia University, Glenside, PA USA; 6 Department of Biochemistry & Molecular Biology, Sidney Kimmel Cancer Center, Thomas Jeferson University, Philadelphia, PA 19107 USA; 7 Ecological and Evolutionary Signal-Processing and Informatics Laboratory, Department of Electrical and Computer Engineering, College of Engineering, Drexel University, Philadelphia, PA 19104 USA; 8 Department of Cancer and Cellular Biology, Lewis Katz School of Medicine, Philadelphia, PA 19140 USA; 9 Department of Clinical Genetics, Fox Chase Cancer Center, 333 Cottman Avenue, Philadelphia, PA 19111 USA; 10 Department of Surgical Oncology, Fox Chase Cancer Center, Philadelphia, PA 19111 USA; 11 Department of Radiation Oncology, Fox Chase Cancer Center, Philadelphia, PA 19111 USA

**Correction: BMC Genomics 24, 212 (2023)**

**https://doi.org/10.1186/s12864-023-09310-8**

Following publication of the original article [1], it was reported that supplementary tables 1, 2 and 8 were missing from the published article. Additionally, the incorrect versions of Figs. 1 and 4 were published. The updated figures and supplementary files are included in this Correction and the original article has been updated.

**Fig. 1:**
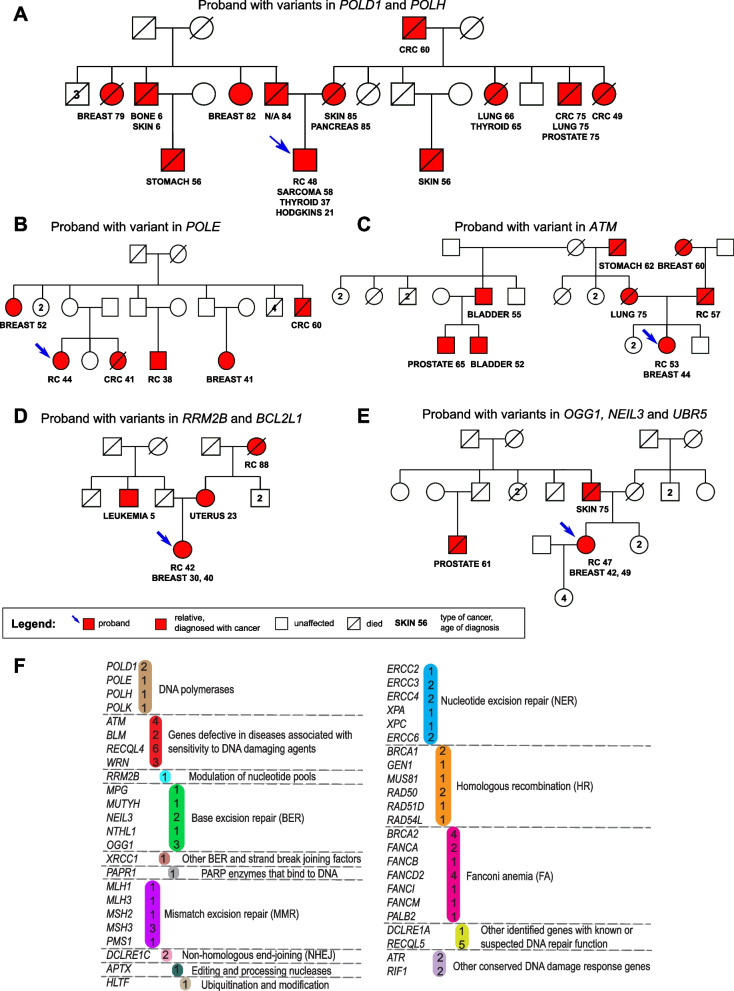
Select pedigrees from the eoRCC patient cohort and enrichment of predicted pathogenic variants in DNA repair genes in the cohort. **A**-**E**. Pedigrees of eoRCC patients with variants in: **A**—POLD1 and POLH; **B**—POLE; **C**—ATM; **D**—RRM2B and BCL2L1; **E**—OGG1, NEIL3 and UBR5. **F**. Summary of variants in genes and pathways, identifed in the cohort. In color—number of variants identifed for each gene. For detailed information, see Supplementary tables 1 and 2

**Fig. 4:**
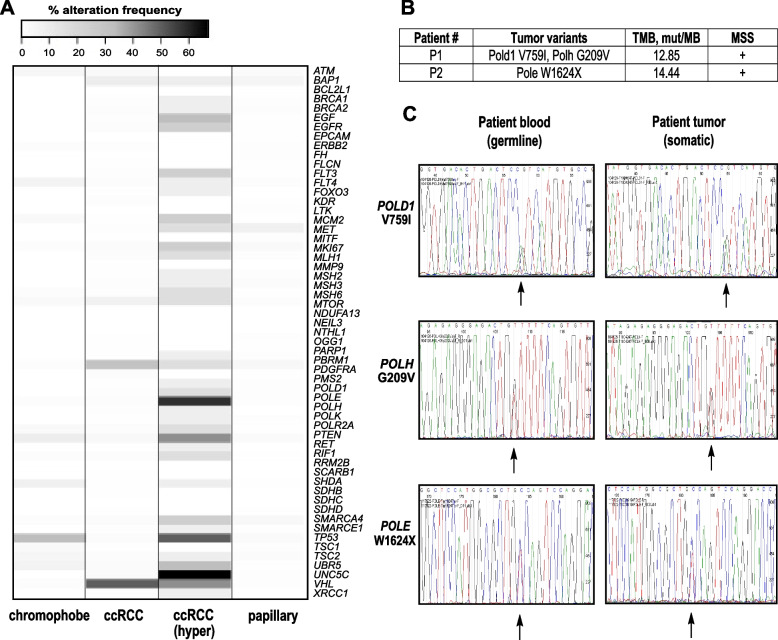
Renal tumors carrying polymerase variants showed high TMB, MSS, and no LOH. **A**. Percent alteration frequency in 897 tumors from TCGA in diferent histological types of RCC: chromophobe (*n* = 66), ccRCC—clear cell renal cell carcinoma (*n* = 538), ccRCC (hyper)—hypermutated samples (*n* = 12), papillary (*n* = 293). **B**. TMB and MSS data are presented for Pt #1 (POLD1 V759I, POLH G209V) and Pt #2 (POLE W1624X). **C**. Tumor and normal Sanger sequencing for variants in Pt #1 (POLD1 V759I, POLH G209V) and Pt #2 (POLE W1624X) showing no LOH. Arrows show variants of interest on sequencing tracks

## Supplementary Information

**Additional file 1: Supplementary table 1.** List of candidate genes for WES analysis. **Supplementary table 2.** Annotation of candidate variants identified in the 22 eoRCC patients.

**Additional file 2: Supplementary Table 8.**
