# Supplementary material for: Candidate variants in DNA replication and repair genes in early-onset renal cell carcinoma patients referred for germline testing
Source: BMC Genomics. 2023 Apr 24;24:212. doi: 10.1186/s12864-023-09310-8 (PMC10123997; doi:10.1186/s12864-023-09310-8)
Supplement: Supplementary file 1 — Additional file 1: Peripheral blood lymphocyte DNA analysis: whole exome sequencing, gene variants annotation and prioritization. Supplementary Figure 1. Pathway enrichment for the genes with identified DDR germline variants according to over-representation analysis (ORA). FDR is provided inside the boxes of the heat map. Supplementary Figure 2. A. siRNA depletion of POLD1, POLE, POLH, POLK, RRM2B and ATM genes in Caki RCC cell line. A. Cells were transfected with the designated siRNAs (two per gene), or GL2 control or WRN positive control. Cells were fixed, permeabilized, blocked and stained for γH2AX antibody. Cells were scored for γH2AX foci and the data are plotted as relative induction of γH2AX to GL2 control from 2 independent experiments. *** for p<0.001, ** for p<0.01, * for p<0.05 and NS for p>0.05, Wilcoxon signed rank test. Supplementary Figure 3. Representative cell cycle data from immortalized B-cell lines from Pt #1 (POLD1, POLH), Pt#2 (POLE), Pt#16 (POLK) PBMCs and corresponding immortalized B-cell lines from matched control. A. Percent positive gated cells is presented for each cell cycle phase for each cell line (controls on the left in blue, cases on the right in red). Data for 3 independent repeats are presented. *** for p<0.001, ** for p<0.01, * for p<0.05, NS for p>0.05, Wilcoxon signed rank test. Supplementary Figure 4. Difference in DNA replication fork elongation/restoration in EBV-transformed cell line with POLK (Pt #16) variant, and representative pictures of DNA fibers for the main figures 2E and 2F. For all graphs: *** for p<0.001, ** for p<0.01, * for p<0.05 and NS for p>0.05, unpaired, non-parametric t-test, Mann-Whitney criteria. Means with SD are plotted. Data for 3 independent repeats are presented as IdU tract length or CldU/IdU tract length ratio. A. Difference in DNA replication fork elongation/restoration in EBV-transformed cell line with POLK E29K variant at the baseline and replication stress was assessed using DNA fiber assay. [file 12864_2023_9310_MOESM1_ESM.zip › 12864_2023_9310_MOESM1_ESM.pdf]

Supplementary Text.

Supplementary Results.

We analyzed oncogenic drivers in tumors from Pt #1 and Pt #2 and found higher copy number variants in genes that are typically associated with RCC and/or have been associated with higher mutation loads **(Supplementary Table 8)** [1-3]. However, typically it is mutations, and not copy number variants, in these genes that have been associated with RCC and/or higher mutation loads. It is possible that these alterations may be the result of the mutator phenotype due to the polymerase candidate variants observed and/or these alterations contributed to the hypermutator phenotype.

Supplementary Methods.

Peripheral blood lymphocyte DNA analysis: whole exome sequencing, gene variants annotation and prioritization.

Whole exome sequencing was performed by BGI Americas Corporation (Cambridge, MA, USA) at 100× average coverage. Agilent SureSelect XT All Exon V6 kit was used for exon capture (Agilent Technologies, Wilmington, DE, USA). Library preparations were done using Illumina standard protocol. Each captured library was indexed, then loaded onto Hiseq2000 platform (Illumina, Hayward, CA, USA) for 100 bp paired-end high-throughput sequencing. Sequence reads were mapped to human reference genome (hg19) using the Burrows-Wheeler Aligner (BWA) [4]. Single Nucleotide Polymorphisms (SNPs) and small Insertion/Deletions (InDels) were detected using Genome Analysis Toolkit (GATK) [5].

As previously described [6], selected annotations were applied to filter and prioritize the variants prior to manual evaluation. (a) Hard-filtering step to exclude variants highly unlikely to be causative. Variants were required to have genotype quality ≥10, read depth ≥10, and maximum population frequency <0.01. (b) Variants were required to be predicted to impact the protein sequence derived from any RefSeq transcript, or to be previously reported as pathogenic or likely pathogenic by at least one of the variant effect prediction tools (SIFT, POLYPHEN2, LRT, MutationTaster, MutationAssessor, or CADD) or reported in ClinVar [6, 7]. (c) All candidate variants were extremely rare as determined by examination of representation in the Genome Aggregation Database (gnomAD) [8]. (d) As previously described [6], for a candidate gene approach, datasets of RCC-related genes obtained from multiple sources, both commercial and publicly available were integrated. RCC-related genes were obtained from Ingenuity [9], TCGA (using cBioPortal [10-13]), DisGeNet [14], ICGC [15], HGMD [16], GeneCards [17], and OMIM [18] Professional databases; genes implicated in multiple cancers (cancer census genes) were obtained from COSMIC database [12] and our previous publication [19, 20].

The candidate gene list was developed by a comprehensive hypothesis-driven framework with the following assumptions: **1)** genes involved in genome stability (using Gene Ontology terms such as DNA repair, DNA replication, DNA damage checkpoints, cell cycle, mitotic machinery, replication stress, DNA damage response, chromatin remodeling) would be important for general hereditary cancer risk [21-24], and **2)** an expanded network of genes relevant to renal biology (such as cellular metabolism) and renal cancer [21, 23, 24].

Subsequently, the gene candidates were prioritized by building a network using Cytoscape program and MiMi plugin, and subsequently only retaining the genes that were either nominated by two or more sources or nominated by one database but interacting with two or more high-confidence genes. **Supplementary Table 1** summarizes the genes in the candidate gene list (n=613). All prioritized gene variants were confirmed by examining the exome reads for sequence quality and variant representation in gnomAD[8] and/or dbSNP, and variants of interest by direct Sanger sequencing.

WebGestalt tool was used for gene set enrichment analysis to extract biological insights from the genes of interest [25]. The online WebGestalt tool was used, and an over representation analysis was performed. The results reported are after correcting for multiple testing (FDR threshold at 0.01 was applied to the results). Finally, MSI or MSS (from clinical PCR-based testing and/or IHC-testing for mismatch repair proteins) was extracted from the patient's clinical data.

Tumor whole-exome sequencing.

DNA extraction from FFPE was performed by following standard protocols [6]. Briefly, tumor cells were collected from an H&E-stained section that was demarcated by a pathologist. The cells were deparaffinized by incubation with xylene at 56°C for 1 hr. Xylene was removed and the cells were washed with descending concentrations of ethanol and the pellet was allowed to dry at 56°C for 10 min. This was followed by DNA isolation using the QIAmp DNA micro kit (Qiagen catalog number 56304). The isolated DNA was measured on Nanodrop and used for further experiments. Whole-exome sequencing was performed by BGI Genomics (Cambridge, MA) at 100x average coverage. BGI Genomics performed the sample quality control, library construction, and sequencing. Briefly, Agilent SureSelect XT All Exon V6 kit was used for exon capture (Agilent Technologies, Wilmington, DE, USA). Library preparations were done using Illumina standard protocol. Each captured library was indexed, then loaded onto Hiseq2000 platform (Illumina, Hayward, CA, USA) for 100 bp paired-end high-throughput sequencing. DNA sequence reads were aligned, and bad reads were removed as described in [6, 26]. This aligned sequence data was analyzed for somatic mutation rates using the common guidelines that have been recently published [27]. TMB was measured by counting all non-synonymous missense mutations found per tumor that have not been previously annotated as germline. For this, variant calling/filtering, and annotation were performed on the aligned sequence data using the publicly available GATK Best Practices [5], and Annovar [28]. The filters applied for processing the genomic data will be as follows: common polymorphisms are removed by filtering against available matched normal from each patient, and GATK recommended publicly available Reference Genome resources such as dbSNP [29], the 1000 genomes project [30] the International Hapmap project [31]. Additionally, after annotation, variants with allele frequencies greater than 0.05 were filtered out by publicly available gnomAD frequency data [32].

Analysis of RCC tumor studies in TCGA

cBioPortal (http://www.cbioportal.org) was used to access data for the candidate genes (*ATM, BCL2L1, BRCA1, BRCA2, EGF, EGFR, ERBB2, FH, FLT3, FLT4, FOXO3, KDR, LTK, MCM2, MKI67, MMP9, MSH3, MTOR, NDUFA13, NEIL3, NTHL1, NXF1, OGG1, PARP1, PBRM1, PDGFRA, POLD1, POLE, POLH, POLK,*

POLR2A, RET, RIF1, RRM2B, SCARB1, SDHB, SMARCA4, SMARCE1, TSC2, UBR5, UNC5C, XRCC1, BAP1, FH, FLCN, MET, MITF, PTEN, SDHA, SDHB, SDHC, SDHD, TSC1, TSC2, VHL, TP53, MLH1, MSH2, PMS2, EPCAM, MSH6) from the most recent TCGA studies (data downloaded in December 2019). The studies used were Kidney Chromophobe (n = 66), Kidney Renal Clear Cell Carcinoma (n = 538), Kidney Renal Papillary Cell Carcinoma (n = 293). All the studies were TCGA Firehouse Legacy datasets. For the analysis, we calculated the TMB in each sample. Mutation counts were adjusted by dividing each result by candidate gene length. These samples were analyzed separately. Data has been processed using RStudio and GraphPad Prism 9 (https://www.graphpad.com/) .

Modelling of DNA polymerase variant interactions

The structural information on Pol ε family of proteins comes mainly from yeast structures found in the PDB, obtained by both X-ray diffraction and cryo-EM methods. The X-ray diffraction structure of the N-terminal lobe in the presence of DNA (PDB code: 4M8O)[33] provided high resolution data for the preliminary model of the human protein discussed here (using Swiss-Model[34]). The more recent cryo-EM structure of the entire yeast Pol2 (analogue for human Pol ε) in complex with the other subunits (PDB code: 6WJV)[35] provided a lower resolution template for modeling the whole human Pol ε with a view to portions that interact with the other subunits of the holoenzyme. There was also a structure of the human Pol ε C-terminal residues 2142 to 2286 (PDB code: 5VBN)[36] that is in complex with the B-subunit of the holoenzyme. To refine our homology models, we captured the best structural data available for each portion of Pol ε and generated two models of the human protein in the presence and absence of DNA. Model refinement methods Rosetta Relax and SCWRL4[37, 38] were used to optimize side chain rotamer interactions with other subunits, substrate DNA and incoming NTP's. UCSF Chimera[39] was used to align models and published structures, to assess residue contacts and hydrogen bonding, and to rationalize mechanistic effects of novel variants on a variety of protein functions, including interactions with other subunits in the holoenzyme.

DNA-bound polymerase structures for PolD1 and Pol η structure motifs was generated by I-TASSER[40-42], then refined in PYMOL (http://www.pymol.org) and then further refined based on the results of the SWISS-model[34] and by using the most homologous structures in the PDB. For PolD1, *Saccharomyces cerevisiae* (PDB code: 3IAY) structure and for Pol η *Saccharomyces cerevisiae* (PDB code: 4O3P) was used. For PolD1 the refined structure shown is from amino acid residues 96-985. The colored functional motifs are exonuclease (residues 130-477) and the polymerase domain (residues 550-978). Protein domain regions were based on pfam (http://pfam.xfam.org/family/PF00136).

Known cancer drivers and novel variants in PolD1 and Pol ε were mapped on to the respective homology models. Pol ε whole-length model was used to represent novel variants, found in C-terminal domain, and shorter model was used to represent location of variants when binding DNA. Novel Pol ε and PolD1 variants were obtained from kidney cancer studies in TCGA (http://www.cbioportal.org).

Predicting the change in stability of missense variants

In order to assess the effects of missense variants on protein stability, we used the Rosetta Molecular Modeling Suite module named "ddg-monomer"[43, 44] (meaning ddG of the variant relative to the wild type amino acid). For this module to be a reliable method of predicting stability changes, the input protein structure model should be complete with no chain breaks. As the initial models built from various model organism templates contain gaps and incomplete loops, we chose to utilize AlphaFold2 models for each of the Pol ε, PolD1, and Pol η. The company DeepMind has made structure predictions of the entire human proteome freely available from an EMBL-EBI server (https://alphafold.ebi.ac.uk/). Prior to using the ddg-monomer module, each of the alphafold models were minimized with required constraints by another Rosetta minimization tool.

The purpose of the ddg-monomer module is to predict the change in stability (the ddG) of a monomeric protein induced by a point mutation[45]. The application takes as input the pre-minimized wild-type model and generates a structural model of the point-mutant. The ddG is given by the difference in Rosetta energy between the wild-type structure and the point mutant structure. The application was run using the high-resolution protocol (using the current Rosetta Scoring Function [beta_nov15] and for specific settings see the application file in **Supplementary Table 7**), and 25 models each of the wild-type and mutant structures were generated, and the most accurate ddG is taken as the difference between the mean of the top-3-scoring wild type structures and the top-3-scoring point-mutant structures. Rosetta follows the convention that negative ddG values indicate increased stability i.e., ddG = mutant energy - wildtype energy.

The ddg-monomer application was used to predict stability changes for the PolD1 V759I variant, Pol η G209V variant, 20 different PolD1 variants, and 23 Pol ε variants from TCGA (see **Supplementary Tables 4 and 5**). The change in stability of the variant relative to the wildtype residue is listed in the ddG column, and the color coding is from green (stabilizing) to white to red (destabilizing). It should be noted that the Alphafold models are apoprotein structures and the Rosetta ddg-monomer calculations are performed without consideration of DNA or metal ligands. While there are minor shifts in the position of a few secondary structure elements, the change in stabilities calculated on the apoprotein models are equivalent to variants in the homology model with DNA (For example when comparing the Alphafold PolD1 apoprotein model to a homology model built in the presence of DNA substrate, the RMSD between 586 alpha carbon atom pairs is 1.16 square angstroms).

Protein expression and purification

Polymerase delta complex expression vectors were obtained from Dr. Pomerantz at the Thomas Jefferson University in Philadelphia. Vectors information could be found in[46]. Pol η expression plasmid was purchased from GenScript (Piscataway, NJ), and contains the cDNA sequence corresponding to 1-711 amino acid inserted into a pET32a backbone. QuikChange Lightning Multi Site-Directed Mutagenesis Kit (Agilent Technologies, CA) was used for site-directed mutagenesis, changes were confirmed by sequencing performed by Genewiz (South Plainfield, NJ). BL21-CodonPlus(DE3)-RIL competent cells (Agilent Technologies, CA) were used as expression strain. Pol δ wild type (wt) and Pol δ containing the PolD1 V759I variant protein complexes were expressed and purified as described[46] before with minor changes. Briefly, cell lysis was performed by 10

cycles with microfluidizer at the 10-15 psi pressure. Pol η wt and Pol η G209V variant were expressed and purified as described[47, 48] with minor change: instead of MonoS column and buffer C, dialysis was performed before concentration with 2 L of the following buffer (25 mM sodium phosphate (pH 7.4), 10% glycerol, 200 mM NaCl, 5 mM β-mercaptoethanol). Identified protein fractions were analyzed by SDS-PAGE gels. Selected fractions containing highly purified polymerase were aliquoted and frozen and stored at −80 °C until further use.

Primer extension, single nucleotide incorporation and 8-oxoG DNA lesion bypass.

The reactions were performed at 37°C with 20 nM Cy3/5-labeled oligonucleotide substrates (see **Supplementary Table 6)**, 25 mM Tris-HCl (pH 8.8), 1 mM DTT, 0.01% Igepal, 0.1 mg/ml BSA, 10% glycerol, 10 mM $MgCl_2$, 20 nM of enzyme (Pol δ wild type complex or Pol δ PolD1 V759I complex, Pol η wild type or Pol η G209V), and 500 µM dNTPs[47, 49, 50]. Reactions were terminated by adding 10 µl of stop solution (45 mM Tris-HCl, 45 nM Boric acid, 1 mM EDTA, 6% Ficoll Type 400, 3,5 M Urea, 0.005% Xylene Cyanol). The products were separated by electrophoresis in a 15% denaturing polyacrylamide gel (Invitrogen, MA), and detected by PharosX Plus Imager (BioRAD, CA) and quantified using ImageJ (https://imagej.nih.gov/ij/) and GraphPad Prism 8 software. All substrates were purchased as duplexes from Integrated DNA Technologies (Coralville, IA, USA) and were purified via HPLC. All dNTPs were purchased from standard suppliers (Thermo Fisher Scientific, cat. #R1121) which are typically of >99% purity. These are standard commercially available reagents used in biochemical activity assays [51, 52].

Assays with primary PBMCs culture, patient- or control-derived EBV cell lines and RCC cell lines.

DDR assays were performed using the primary PBMCs as previously described[19, 20]. Primary PBMCs were available from 20 of the exome-sequenced patients and 20 age-matched and gender-matched individuals without a cancer diagnosis or a family history of cancer. All PBMCs were obtained from the FCCC Biosample Repository Facility. Briefly, cells were cultured in RPMI-1640 containing 15% fetal bovine serum (HyClone Laboratories, Logan, UT), 2 mM L-glutamine (Life Technologies, Grand Island, NY), 50 µM 2-mercaptoethanol (Sigma-Aldrich, St. Louis, MO), 0.2 units human recombinant insulin (Sigma-Aldrich, St. Louis, MO) per ml, 50 units penicillin and 50 mg streptomycin per ml (complete RPMI), and then stimulated with phytohemagglutinin (PHA)-M (Life Technologies, Grand Island, NY) and recombinant human interleukin 2 (IL-2) (NCI Preclinical Repository) for 72 h. For immunofluorescence, cells were allowed to attach to poly-d-lysine-coated 96-well plates, and then treated with vehicle, or 20 µM aphidicolin, and fixed in paraformaldehyde 2 hours later, permeabilized, blocked and then stained with anti-γH2AX antibody (#05–636, Millipore, Temecula, CA). After the primary antibody, cells were stained with the secondary anti-mouse antibody (Cell Signaling Technology Inc., Danvers, MA) and counter stained with DAPI. Cells were imaged on the 6 ImageXpress Micro automated microscope (Molecular Devices, Sunnyvale, CA) and analyzed by MetaXpress software. Foci were scored for γH2AX staining, and the results were displayed and exported using the AcuityXpress software package (Molecular Devices, Sunnyvale, CA).

Protein expression studies were performed using EBV-immortalized cell lines from patient and matched controls. Briefly, whole cell lysates were prepared, and Western blot analysis was performed for the respective

proteins and β-actin or GAPDH loading control. Total protein levels were quantified and normalized to the loading control. Primary antibodies used were: PolD1 (ab186407, Abcam, Cambridge, MA), Pole (#MA5-13616, Thermo Fisher Scientific, Waltham, MA), Pol eta (#13848, Cell Signaling Technology Inc., Danvers, MA), Pol kappa (A301-975A-T, Santa Cruz Biotechnology Inc., CA), β-actin (ab20272, Abcam, Cambridge, MA), GAPDH (from Loading Control Ab sampler kit, #5142T, Cell Signaling Technology Inc., Danvers, MA).

For cell cycle analysis, immortalized EBV cells were fixed in 96% ethanol at $1x10^6$ cells. Then cells were washed in PBS and finally stained with PI/RNAse Staining buffer (cat. #550825, BD Biosciences, NJ). The Guava PCA (Millipore, IL) for cell cycle analysis.

All cell lines used in the study were authenticated by IDEXX BioAnalytics. For siRNA studies, Caki cells were plated in 96-well plates and then transfected with GL2 (negative control), WRN (positive control) or two independent siRNAs (5nM) for each protein. Cells were then fixed in paraformaldehyde, permeabilized, blocked and then stained with anti-γH2AX antibody (#05–636, Millipore, Temecula, CA). After the primary antibody, cells were stained with the secondary anti-mouse antibody (Cell Signaling Technology Inc., Danvers, MA) and counter stained with DAPI. Cells were imaged on the 6 ImageXpress Micro automated microscope (Molecular Devices, Sunnyvale, CA) and analyzed by MetaXpress software. Foci were scored for γH2AX staining, and the results were displayed and exported using the AcuityXpress software package (Molecular Devices, Sunnyvale, CA). The data are plotted as relative induction of γH2AX to GL2 control from 2 independent experiments.

Cellular viability experiments were performed using the EBV lines from patient and matched controls. Here, cells were plated in 96-well plates and were treated for 2h with vehicle (cell culture medium) or with aphidicolin (Sigma-Aldrich, St. Louis, MO), or Ultraviolet (UV) light at concentrations or doses shown respectively. At 72h post-treatment, CellTiterBlue (#G8080, Promega Corporation, Madison, WI) was added and the absorbance was read using a Perkin Elmer Plate Reader (PerkinElmer Inc, Waltham, MA). The signal was read using a Perkin Elmer Plate Reader.

DNA fiber assay was performed as previously described[53, 54]. Briefly, patient-derived EBV cell lines carrying original variants in DNA polymerase (Pt #1 - PolD1 V759I/Pol η G209V, Pt #2 – Pol ε W1624X, P t#16 – Pol κ E29K, see **Table 1**) were labelled with 250 µM of 5-Iodo-2′-deoxyuridine or IdU (Sigma-Aldrich, MO) or/and 50 µM of 5-Chloro-2'-deoxyuridine or CldU based on experimental conditions (see **Figure 2** and **Supplementary Figure 4**). Post labeling, the cell lines were pelleted, re-suspended in ice cold PBS buffer (~3000 cells/µl), then 2.5 µl cells (~ 9000 cells) were spotted onto a glass slide (Superfrost Plus, ThermoFisher Scientific, Waltham, MA), and mixed with 7.5 µl of lysis buffer (200 mM Tris-HCl pH7.4, 50 mM EDTA, 0.5% SDS). For DNA fibers, cells were lysed for 8 min and then the slides were tilted at 45° angle and air-dried. DNA fibers on the slide were fixed with a methanol: acetic acid (3:1) solution for 10 min, and then slides were rinsed with water, air-dried and frozen overnight. Next day, the DNA fiber slides were washed with PBS, DNA was denatured using 2.5 M HCl for 2.5 h, washed in PBST (PBS & 0.1% Tween-20), and then blocked with 2% BSA for 40 min at RT. After blocking, the fibers slides were incubated with anti-IdU/anti-CldU antibody (1:100, cat. # 347580, BD Biosciences, NJ & 1:500, cat. #NB500-169, Novus Biologicals, CO, correspondingly) followed by a

secondary antibody (1:300, cat. #A-11062 anti-mouse Alexa594 & cat. #A-11006 anti-rat Alexa488, ThermoFisher Scientific, Waltham, MA). The slides were mounted using ProLong Diamond Antifade Mountant (cat. # P36970, ThermoFisher Scientific, Waltham, MA) and imaged using the Leica SP8 (Leica Microsystems Inc., Buffalo Grove, IL) and Nikon TS2R Inverted Microscope (Nikon Instruments Inc., Melville, NY) microscopes, and the data were analyzed using the ImageJ & GraphPad Prism 9 software.

Mutational Signature Analysis.

We used signature refitting to identify mutational processes by fitting the mutational signatures published in the COSMIC catalog [55-57] to the mutational profiles of the single base substitutions (SBS) and doublet base substitutions (DBS) from tumor sequencing data of Pt 1 with *POLD1*/*POLH* variants, and Pt 2 with *POLE* variant. We aggregated refitting results using our in-house package, MetaMutationalSigs, which outputs results from high-performing packages [58]. We chose to report results from Sigflow as this tool reported the most consistent signatures [59].

Analysis of somatic drivers.

To analyze somatic drivers in the tumor, we used the OncoKB™ cancer gene list (https://www.oncokb.org/cancerGenes). The list of cancer genes was applied to tumor sequencing data from Pt 1 with *POLD1*/*POLH* variants, and Pt 2 with *POLE* variant. Briefly, all somatic mutations were filtered by population frequency of < 1% in 1000 Genome Project normal population and dbSNP database. For somatic SNVs/SNPs, only non-synonymous variants which were predicted to have moderate or high impact on protein function by BGI variant annotation tool in the exonic region or splicing sites were retained. For small insertions and deletions, we only kept disruptive in-frame insertions or deletions and frame-shifting insertions and deletions. For CNV analysis, we only focused on exonic and spice site regions which have at least 2-fold copy ratio changes.

Statistics

Where appropriate we used unpaired or paired non-parametric T-test, Mann-Whitney, or Wilcoxon criteria for the assessment of the differences between cases and controls in γH2AX immunofluorescent staining foci assessment, protein expression, cell counts, cell cycle analysis, CTB assay, DNA fiber assay and biochemical assays. P-value less 0.05 was considered significant, *** for $p<0.001$, ** for $p<0.01$, * for $p<0.05$ and NS for $p>0.05$.

Supplementary References.

1. Li L, Rao X, Wen Z, Ding X, Wang X, Xu W, Meng C, Yi Y, Guan Y, Chen Y *et al*: **Implications of driver genes associated with a high tumor mutation burden identified using next-generation sequencing on immunotherapy in hepatocellular carcinoma**. *Oncol Lett* 2020, **19**(4):2739-2748.
2. Ma K, Huang F, Wang Y, Kang Y, Wang Q, Tang J, Sun P, Lou J, Qiao R, Si J *et al*: **Relationship between tumor mutational burden, gene mutation status, and clinical characteristics in 340 cases of lung adenocarcinoma**. *Cancer Med* 2022, **11**(22):4389-4397.

3. Smolle E, Leithner K, Olschewski H: **Oncogene addiction and tumor mutational burden in non-small-cell lung cancer: Clinical significance and limitations**. *Thorac Cancer* 2020, **11**(2):205-215.
4. Li H, Durbin R: **Fast and accurate short read alignment with Burrows-Wheeler transform**. *Bioinformatics* 2009, **25**(14):1754-1760.
5. McKenna A, Hanna M, Banks E, Sivachenko A, Cibulskis K, Kernytsky A, Garimella K, Altshuler D, Gabriel S, Daly M *et al*: **The Genome Analysis Toolkit: a MapReduce framework for analyzing next-generation DNA sequencing data**. *Genome Res* 2010, **20**(9):1297-1303.
6. Nicolas E, Demidova EV, Iqbal W, Serebriiskii IG, Vlasenkova R, Ghatalia P, Zhou Y, Rainey K, Forman AF, Dunbrack RL, Jr. *et al*: **Interaction of germline variants in a family with a history of early-onset clear cell renal cell carcinoma**. *Mol Genet Genomic Med* 2019, **7**(3):e556.
7. Landrum MJ, Lee JM, Benson M, Brown GR, Chao C, Chitipiralla S, Gu B, Hart J, Hoffman D, Jang W *et al*: **ClinVar: improving access to variant interpretations and supporting evidence**. *Nucleic Acids Res* 2018, **46**(D1):D1062-d1067.
8. Karczewski KJ, Francioli LC, Tiao G, Cummings BB, Alföldi J, Wang Q, Collins RL, Laricchia KM, Ganna A, Birnbaum DP *et al*: **The mutational constraint spectrum quantified from variation in 141,456 humans**. *Nature* 2020, **581**(7809):434-443.
9. Calvano SE, Xiao W, Richards DR, Felciano RM, Baker HV, Cho RJ, Chen RO, Brownstein BH, Cobb JP, Tschoeke SK *et al*: **A network-based analysis of systemic inflammation in humans**. *Nature* 2005, **437**(7061):1032-1037.
10. Gao J, Aksoy BA, Dogrusoz U, Dresdner G, Gross B, Sumer SO, Sun Y, Jacobsen A, Sinha R, Larsson E *et al*: **Integrative analysis of complex cancer genomics and clinical profiles using the cBioPortal**. *Sci Signal* 2013, **6**(269):pl1.
11. Cheng WC, Chung IF, Chen CY, Sun HJ, Fen JJ, Tang WC, Chang TY, Wong TT, Wang HW: **DriverDB: an exome sequencing database for cancer driver gene identification**. *Nucleic Acids Res* 2014, **42**(Database issue):D1048-1054.
12. Bamford S, Dawson E, Forbes S, Clements J, Pettett R, Dogan A, Flanagan A, Teague J, Futreal PA, Stratton MR *et al*: **The COSMIC (Catalogue of Somatic Mutations in Cancer) database and website**. *Br J Cancer* 2004, **91**(2):355-358.
13. Joly Y, Dove ES, Knoppers BM, Bobrow M, Chalmers D: **Data sharing in the post-genomic world: the experience of the International Cancer Genome Consortium (ICGC) Data Access Compliance Office (DACO)**. *PLoS computational biology* 2012, **8**(7):e1002549-e1002549.
14. Piñero J, Queralt-Rosinach N, Bravo À, Deu-Pons J, Bauer-Mehren A, Baron M, Sanz F, Furlong LI: **DisGeNET: a discovery platform for the dynamical exploration of human diseases and their genes**. *Database (Oxford)* 2015, **2015**:bav028.
15. International Cancer Genome C, Hudson TJ, Anderson W, Artez A, Barker AD, Bell C, Bernabé RR, Bhan MK, Calvo F, Eerola I *et al*: **International network of cancer genome projects**. *Nature* 2010, **464**(7291):993-998.
16. Stenson PD, Ball EV, Mort M, Phillips AD, Shiel JA, Thomas NS, Abeysinghe S, Krawczak M, Cooper DN: **Human Gene Mutation Database (HGMD): 2003 update**. *Hum Mutat* 2003, **21**(6):577-581.
17. Rebhan M, Chalifa-Caspi V, Prilusky J, Lancet D: **GeneCards: a novel functional genomics compendium with automated data mining and query reformulation support**. *Bioinformatics* 1998, **14**(8):656-664.
18. Hamosh A, Scott AF, Amberger JS, Bocchini CA, McKusick VA: **Online Mendelian Inheritance in Man (OMIM), a knowledgebase of human genes and genetic disorders**. *Nucleic Acids Res* 2005, **33**(Database issue):D514-517.
19. Arora S, Yan H, Cho I, Fan HY, Luo B, Gai X, Bodian DL, Vockley JG, Zhou Y, Handorf EA *et al*: **Genetic Variants That Predispose to DNA Double-Strand Breaks in Lymphocytes From a Subset of Patients With Familial Colorectal Carcinomas**. *Gastroenterology* 2015, **149**(7):1872-1883.e1879.
20. Nicolas E, Arora S, Zhou Y, Serebriiskii IG, Andrake MD, Handorf ED, Bodian DL, Vockley JG, Dunbrack RL, Ross EA *et al*: **Systematic evaluation of underlying defects in DNA repair as an approach to case-only assessment of familial prostate cancer**. *Oncotarget* 2015, **6**(37):39614-39633.
21. Berti M, Vindigni A: **Replication stress: getting back on track**. *Nat Struct Mol Biol* 2016, **23**(2):103-109.
22. Coleman JA, Russo P: **Hereditary and familial kidney cancer**. *Curr Opin Urol* 2009, **19**(5):478-485.
23. Henegan JC, Jr., Gomez CR: **Heritable Cancer Syndromes Related to the Hypoxia Pathway**. *Front Oncol* 2016, **6**:68.

24. Linehan WM, Srinivasan R, Schmidt LS: **The genetic basis of kidney cancer: a metabolic disease**. *Nat Rev Urol* 2010, **7**(5):277-285.
25. Liao Y, Wang J, Jaehnig EJ, Shi Z, Zhang B: **WebGestalt 2019: gene set analysis toolkit with revamped UIs and APIs**. *Nucleic Acids Res* 2019, **47**(W1):W199-w205.
26. Nicolas E, Arora S, Zhou Y, Serebriiskii IG, Andrake MD, Handorf ED, Bodian DL, Vockley JG, Dunbrack RL, Ross EA *et al*: **Systematic evaluation of underlying defects in DNA repair as an approach to case-only assessment of familial prostate cancer**. *Oncotarget* 2015, **6**(37):39614-39633.
27. Merino DM, McShane LM, Fabrizio D, Funari V, Chen SJ, White JR, Wenz P, Baden J, Barrett JC, Chaudhary R *et al*: **Establishing guidelines to harmonize tumor mutational burden (TMB): in silico assessment of variation in TMB quantification across diagnostic platforms: phase I of the Friends of Cancer Research TMB Harmonization Project**. *J Immunother Cancer* 2020, **8**(1).
28. Wang K, Li M, Hakonarson H: **ANNOVAR: functional annotation of genetic variants from high-throughput sequencing data**. *Nucleic Acids Res* 2010, **38**(16):e164.
29. Sherry ST, Ward MH, Kholodov M, Baker J, Phan L, Smigielski EM, Sirotkin K: **dbSNP: the NCBI database of genetic variation**. *Nucleic Acids Res* 2001, **29**(1):308-311.
30. Genomes Project C, Auton A, Brooks LD, Durbin RM, Garrison EP, Kang HM, Korbel JO, Marchini JL, McCarthy S, McVean GA *et al*: **A global reference for human genetic variation**. *Nature* 2015, **526**(7571):68-74.
31. International HapMap C: **The International HapMap Project**. *Nature* 2003, **426**(6968):789-796.
32. Lek M, Karczewski KJ, Minikel EV, Samocha KE, Banks E, Fennell T, O'Donnell-Luria AH, Ware JS, Hill AJ, Cummings BB *et al*: **Analysis of protein-coding genetic variation in 60,706 humans**. *Nature* 2016, **536**(7616):285-291.
33. Hogg M, Osterman P, Bylund GO, Ganai RA, Lundström EB, Sauer-Eriksson AE, Johansson E: **Structural basis for processive DNA synthesis by yeast DNA polymerase ε**. *Nat Struct Mol Biol* 2014, **21**(1):49-55.
34. Waterhouse A, Bertoni M, Bienert S, Studer G, Tauriello G, Gumienny R, Heer FT, de Beer TAP, Rempfer C, Bordoli L *et al*: **SWISS-MODEL: homology modelling of protein structures and complexes**. *Nucleic Acids Res* 2018, **46**(W1):W296-w303.
35. Yuan Z, Georgescu R, Schauer GD, O'Donnell ME, Li H: **Structure of the polymerase ε holoenzyme and atomic model of the leading strand replisome**. *Nature Communications* 2020, **11**(1):3156.
36. Baranovskiy AG, Gu J, Babayeva ND, Kurinov I, Pavlov YI, Tahirov TH: **Crystal structure of the human Polϵ B-subunit in complex with the C-terminal domain of the catalytic subunit**. *J Biol Chem* 2017, **292**(38):15717-15730.
37. Krivov GG, Shapovalov MV, Dunbrack RL, Jr.: **Improved prediction of protein side-chain conformations with SCWRL4**. *Proteins* 2009, **77**(4):778-795.
38. Alford RF, Leaver-Fay A, Jeliazkov JR, O'Meara MJ, DiMaio FP, Park H, Shapovalov MV, Renfrew PD, Mulligan VK, Kappel K *et al*: **The Rosetta All-Atom Energy Function for Macromolecular Modeling and Design**. *Journal of Chemical Theory and Computation* 2017, **13**(6):3031-3048.
39. Pettersen EF, Goddard TD, Huang CC, Couch GS, Greenblatt DM, Meng EC, Ferrin TE: **UCSF Chimera--a visualization system for exploratory research and analysis**. *J Comput Chem* 2004, **25**(13):1605-1612.
40. Yang J, Yan R, Roy A, Xu D, Poisson J, Zhang Y: **The I-TASSER Suite: protein structure and function prediction**. *Nat Methods* 2015, **12**(1):7-8.
41. Roy A, Kucukural A, Zhang Y: **I-TASSER: a unified platform for automated protein structure and function prediction**. *Nat Protoc* 2010, **5**(4):725-738.
42. Zhang Y: **I-TASSER server for protein 3D structure prediction**. *BMC Bioinformatics* 2008, **9**(1):40.
43. Kellogg EH, Leaver-Fay A, Baker D: **Role of conformational sampling in computing mutation-induced changes in protein structure and stability**. *Proteins* 2011, **79**(3):830-838.
44. Caswell RC, Gunning AC, Owens MM, Ellard S, Wright CF: **Assessing the clinical utility of protein structural analysis in genomic variant classification: experiences from a diagnostic laboratory**. *Genome Med* 2022, **14**(1):77.
45. Leaver-Fay A, O'Meara MJ, Tyka M, Jacak R, Song Y, Kellogg EH, Thompson J, Davis IW, Pache RA, Lyskov S *et al*: **Scientific benchmarks for guiding macromolecular energy function improvement**. *Methods Enzymol* 2013, **523**:109-143.

46. Fazlieva R, Spittle CS, Morrissey D, Hayashi H, Yan H, Matsumoto Y: **Proofreading exonuclease activity of human DNA polymerase delta and its effects on lesion-bypass DNA synthesis**. *Nucleic Acids Res* 2009, **37**(9):2854-2866.
47. Beardslee RA, Suarez SC, Toffton SM, McCulloch SD: **Mutation of the little finger domain in human DNA polymerase η alters fidelity when copying undamaged DNA**. *Environ Mol Mutagen* 2013, **54**(8):638-651.
48. Suarez SC, Beardslee RA, Toffton SM, McCulloch SD: **Biochemical analysis of active site mutations of human polymerase η**. *Mutat Res* 2013, **745-746**:46-54.
49. Kent T, Rusanov TD, Hoang TM, Velema WA, Krueger AT, Copeland WC, Kool ET, Pomerantz RT: **DNA polymerase θ specializes in incorporating synthetic expanded-size (xDNA) nucleotides**. *Nucleic Acids Res* 2016, **44**(19):9381-9392.
50. Daee DL, Mertz TM, Shcherbakova PV: **A cancer-associated DNA polymerase delta variant modeled in yeast causes a catastrophic increase in genomic instability**. *Proc Natl Acad Sci U S A* 2010, **107**(1):157-162.
51. Ozdemir AY, Rusanov T, Kent T, Siddique LA, Pomerantz RT: **Polymerase θ-helicase efficiently unwinds DNA and RNA-DNA hybrids**. *J Biol Chem* 2018, **293**(14):5259-5269.
52. Chandramouly G, Zhao J, McDevitt S, Rusanov T, Hoang T, Borisonnik N, Treddinick T, Lopezcolorado FW, Kent T, Siddique LA *et al*: **Polθ reverse transcribes RNA and promotes RNA-templated DNA repair**. *Sci Adv* 2021, **7**(24).
53. Quinet A, Carvajal-Maldonado D, Lemacon D, Vindigni A: **DNA Fiber Analysis: Mind the Gap!** *Methods Enzymol* 2017, **591**:55-82.
54. Nacson J, Krais JJ, Bernhardy AJ, Clausen E, Feng W, Wang Y, Nicolas E, Cai KQ, Tricarico R, Hua X *et al*: **BRCA1 Mutation-Specific Responses to 53BP1 Loss-Induced Homologous Recombination and PARP Inhibitor Resistance**. *Cell Rep* 2018, **24**(13):3513-3527.e3517.
55. Alexandrov LB, Kim J, Haradhvala NJ, Huang MN, Tian Ng AW, Wu Y, Boot A, Covington KR, Gordenin DA, Bergstrom EN *et al*: **The repertoire of mutational signatures in human cancer**. *Nature* 2020, **578**(7793):94-101.
56. Alexandrov Ludmil B, Nik-Zainal S, Wedge David C, Campbell Peter J, Stratton Michael R: **Deciphering Signatures of Mutational Processes Operative in Human Cancer**. *Cell Reports* 2013, **3**(1):246-259.
57. Machado HE, Mitchell E, Øbro NF, Kübler K, Davies M, Maura F, Leongamornlert D, Sanders MA, Cagan A, McDonald C *et al*: **Genome-wide mutational signatures of immunological diversification in normal lymphocytes**. *bioRxiv* 2021:2021.2004.2029.441939.
58. Pandey P, Arora S, Rosen GL: **MetaMutationalSigs: Comparison of mutational signature refitting results made easy**. *Bioinformatics* 2022, **38**(8):2344-2347.
59. Wang S, Tao Z, Wu T, Liu XS: **Sigflow: an automated and comprehensive pipeline for cancer genome mutational signature analysis**. *Bioinformatics* 2021, **37**(11):1590-1592.
60. Campbell BB, Light N, Fabrizio D, Zatzman M, Fuligni F, de Borja R, Davidson S, Edwards M, Elvin JA, Hodel KP *et al*: **Comprehensive Analysis of Hypermutation in Human Cancer**. *Cell* 2017, **171**(5):1042-1056.e1010.

Supplementary Figure Legends.

Supplementary Figure 1. Pathway enrichment for the genes with identified DDR germline variants according to over-representation analysis (ORA). FDR is provided inside the boxes of the heat map.

Supplementary Figure 2. A. siRNA depletion of *POLD1, POLE, POLH, POLK, RRM2B* and *ATM* genes in Caki RCC cell line. A. Cells were transfected with the designated siRNAs (two per gene), or GL2 control or *WRN* positive control. Cells were fixed, permeabilized, blocked and stained for γH2AX antibody. Cells were scored for γH2AX foci and the data are plotted as relative induction of γH2AX to GL2 control from 2 independent experiments. *** for p<0.001, ** for p<0.01, * for p<0.05 and NS for p>0.05, Wilcoxon signed rank test.

Supplementary Figure 3. Representative cell cycle data from immortalized B-cell lines from Pt #1 (*POLD1*, *POLH*), Pt#2 (*POLE*), Pt#16 (*POLK*) PBMCs and corresponding immortalized B-cell lines from matched control. A. Percent positive gated cells is presented for each cell cycle phase for each cell line (controls on the left in blue, cases on the right in red). Data for 3 independent repeats are presented. *** for p<0.001, ** for p<0.01, * for p<0.05, NS for p>0.05, Wilcoxon signed rank test.

Supplementary Figure 4. Difference in DNA replication fork elongation/restoration in EBV-transformed cell line with *POLK* (Pt #16) variant, and representative pictures of DNA fibers for the main figures 2E and 2F. For all graphs: *** for p<0.001, ** for p<0.01, * for p<0.05 and NS for p>0.05, unpaired, non-parametric t-test, Mann-Whitney criteria. Means with SD are plotted. Data for 3 independent repeats are presented as IdU tract length or CldU/IdU tract length ratio. **A.** Difference in DNA replication fork elongation/restoration in EBV-transformed cell line with *POLK* E29K variant at the baseline and replication stress was assessed using DNA fiber assay. At baseline the EBV-transformed cells were labeled with IdU for 20 min, for fork restoration cells then were treated with 100 µM aphidicolin and then labeled with CldU for 40 min. For all conditions, after labeling, cells were lysed, and DNA fibers stretched onto glass-slides, fixed, denatured, blocked, and stained with corresponding antibodies. Fiber images were captured using the Nikon TS2R Inverted Microscope and analyzed in ImageJ software. **B-D.** Additional representative pictures for each DNA fiber experiment: **B** for IdU 20 min labeling (main figures **2E**, **2F,** current figure **A); C** for short aphidicolin treatment (main figures **2E, 2F,** current figure **A**); **D** for treatment with MNNG (main figure **2E**). Scale bar = 5 µm.

Supplementary Figure 5. Pol δ complex primer extension competition assay with quantification. Representative gel image showing reactions performed with 20 nM Cy-3 labeled DNA-duplex template (SA#1), 20 nM of indicated proteins (wild type complex, V759I complex and both wild type + V759 variant complexes in ratio 1:1) and 500 uM dNTPs. Under presence of the both wild type and PolD1 V759I Pol δ complexes, DNA-template was extended less efficiently compared to wild type and more efficiently compared to V759I complexes alone. Data for 3 independent repeats are presented. *** for p<0.001, ** for p<0.01, * for p<0.05 and NS for p>0.05, unpaired, non-parametric t-test, Mann-Whitney criteria.

Supplementary Figure 6. Raw uncropped gel images, corresponding to sub-figures in Figure 2B: **A, B** and **C** for PolD1, Pol η and loading control exposures used to generate the main figure, **D** for Pol ε, **E** for Pol κ.

Supplementary Figure 7. Raw uncropped gel images, corresponding to sub-figures in Figure 3 and Supplementary Figure 5. **A** for Figure 3A for Pol δ complex purification, **B** for Figure 3B Pol η purification, **C** for Figure 3A on right- Pol δ complex primer extension assay, **D** for 3B on right- Pol η primer extension assay, **E** for 3C - Pol η lesion bypass assay, **F** for Supplementary Figure 5.

Supplementary Figure 8. Mutational signature analysis in tumors from Pts 1 and 2. **A.** SBS signatures are reported in tumors from Pts 1 and 2. **B.** DBS signatures are reported in tumors from Pts 1 and 2.

Supplemental Table Legends.

Supplementary table 1. List of candidate genes for WES analysis (Excel file).

Supplementary table 2. Annotation of candidate variants identified in the 22 eoRCC patients (Excel file).

Supplementary table 3. Complete summary of results for the DNA polymerase variants identified in the eoRCC patients.

Supplementary table 4. Protein stability or ddG values for the PolD1 V759I and Pol η G209V variant proteins that were assessed biochemically in the study.

Supplementary table 5. *POLE* and *POLD1* variants in hypermutated ccRCC in TCGA. The table shows the variants from TCGA with allele counts typically observed in the GnomAD database, TMBs observed in association with these variants in other studies [60], and finally protein stability or ddG values for the variant proteins.

Supplementary table 6. DNA substrates used in biochemical assays. The following are the references for the DNA substrates [47, 49, 50]

Supplementary table 7. ddG application file.

Supplementary Table 8. Results from analysis of CNVs, SNVs and Indels using tumor sequencing data from Pt #1 (*POLD1*, *POLH*), and Pt #2 (*POLE*) (Excel file).

A

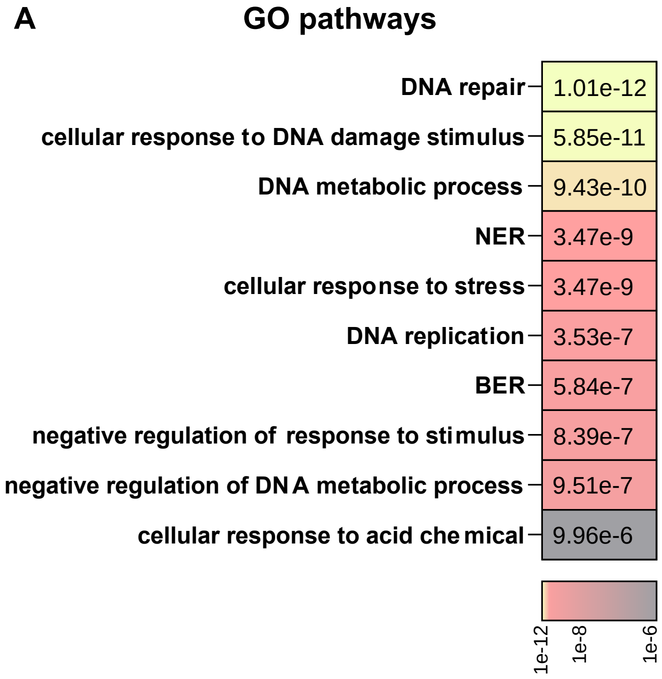

Demidova et al., Supplementary Figure 1

A

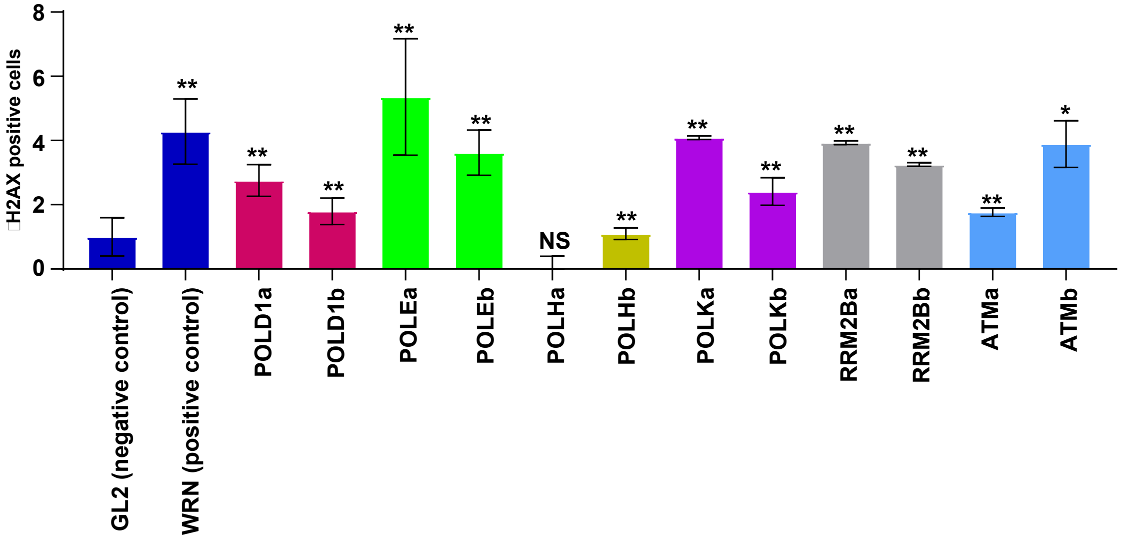

Demidova et al., Supplementary Figure 2

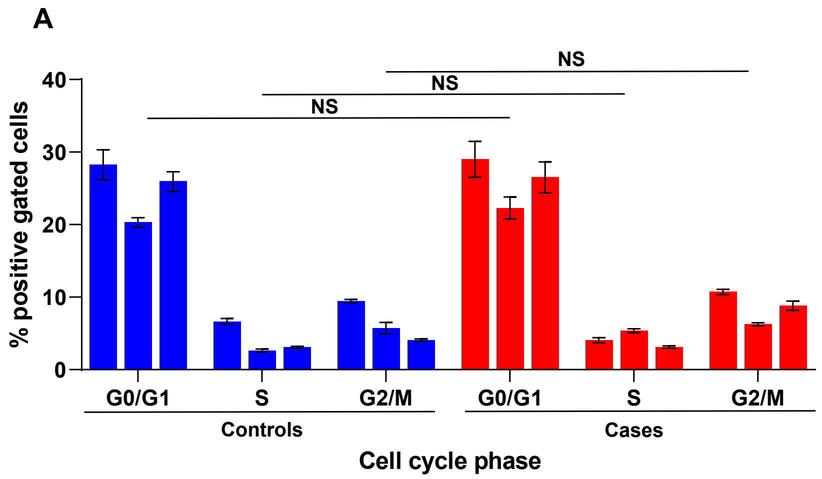

Demidova et al., Supplementary Figure 3

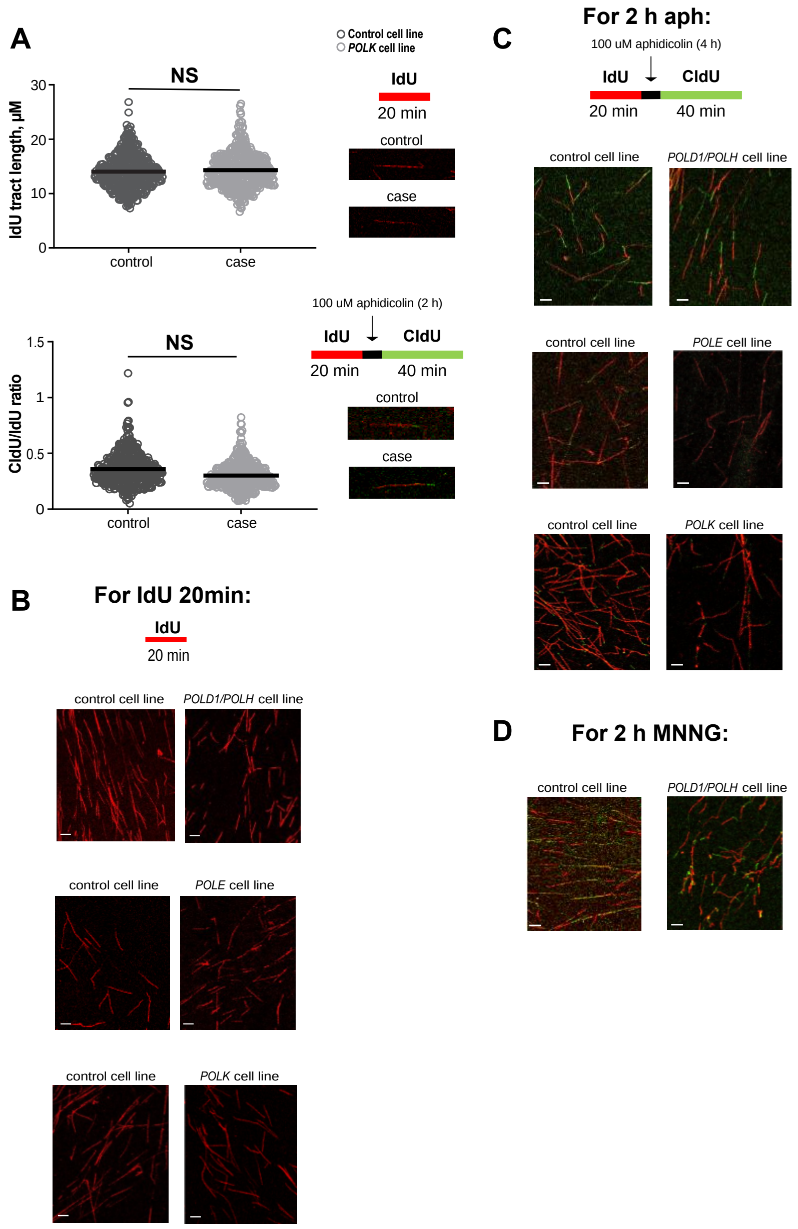

Demidova et al., Supplementary Figure 4

A Pol δ primer extension competition assay

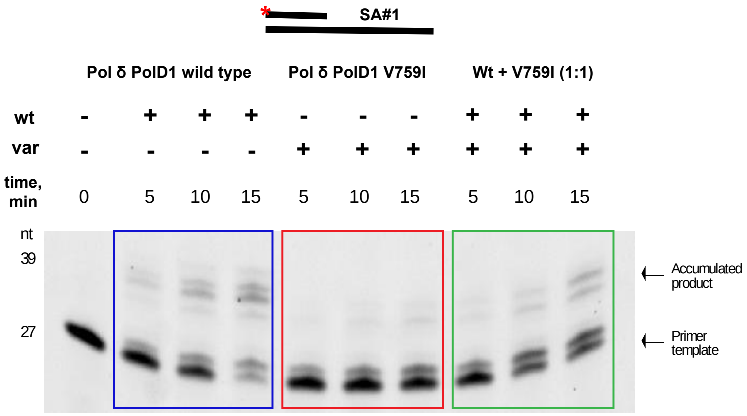

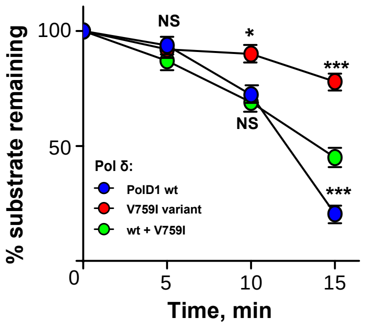

Demidova et al., Supplementary Figure 5

A **For Figure 2B: PolD1**

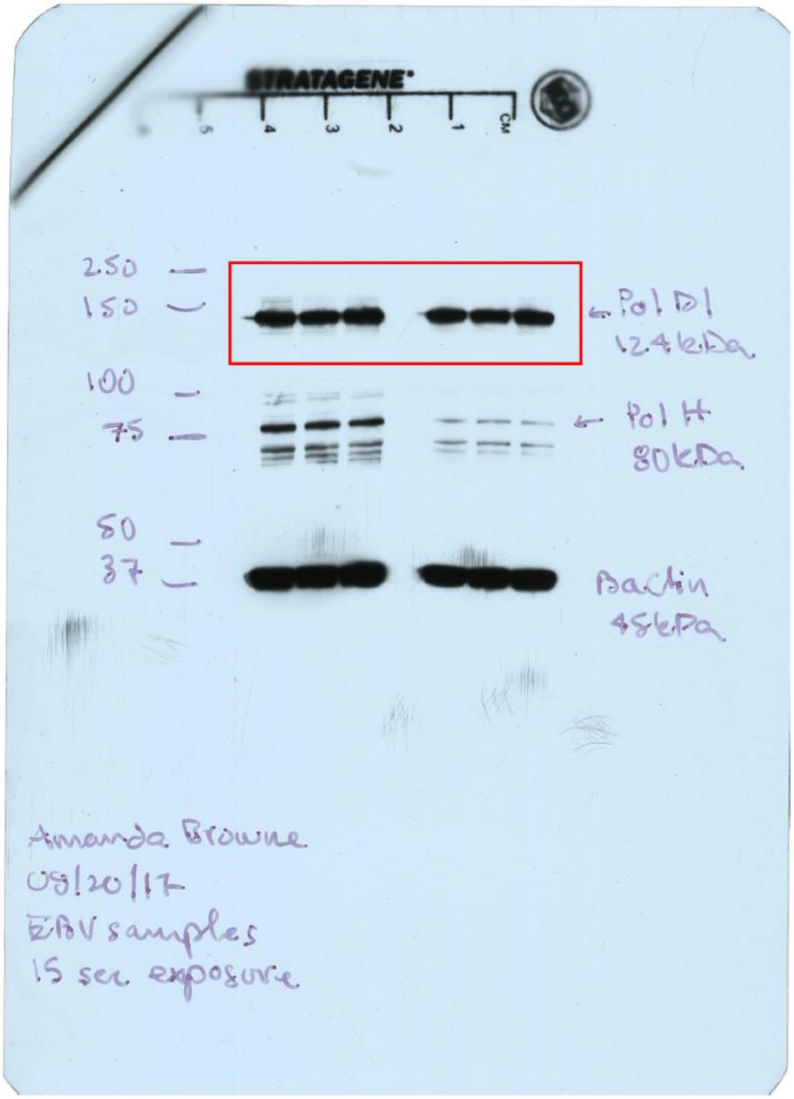

Demidova et al., Supplementary Figure 6

B **For Figure 2B: Polη**

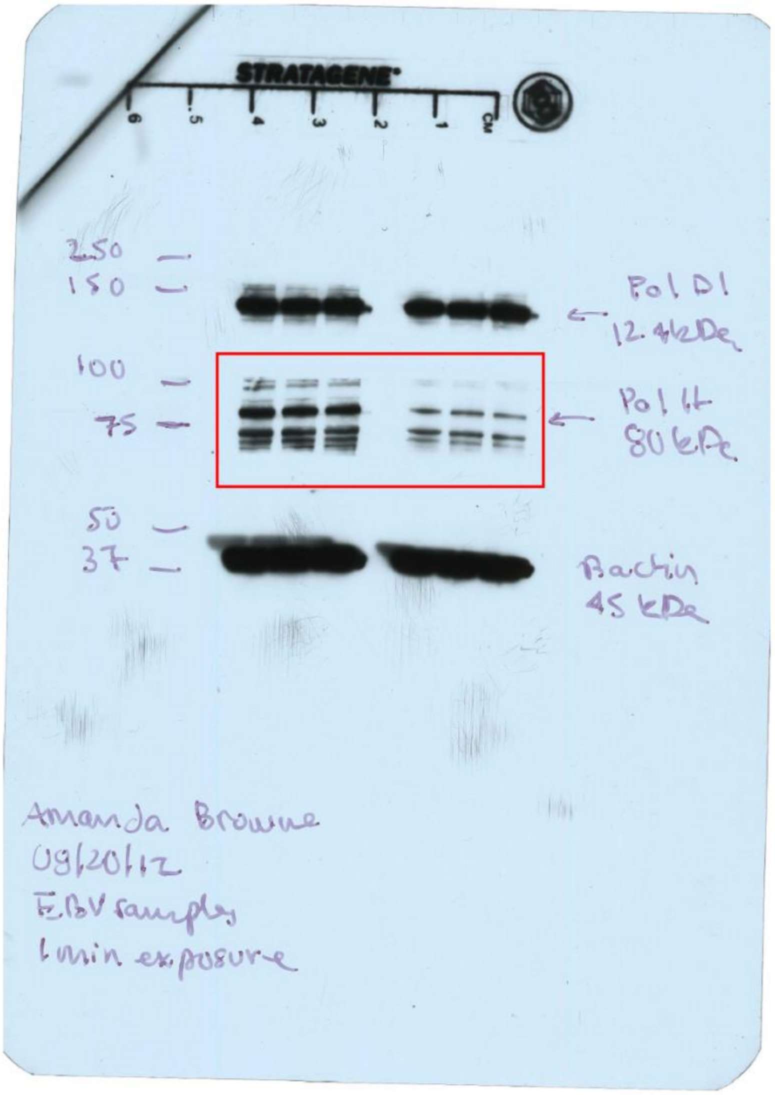

Demidova et al., Supplementary Figure 6

C Figure 2B: Beta actin

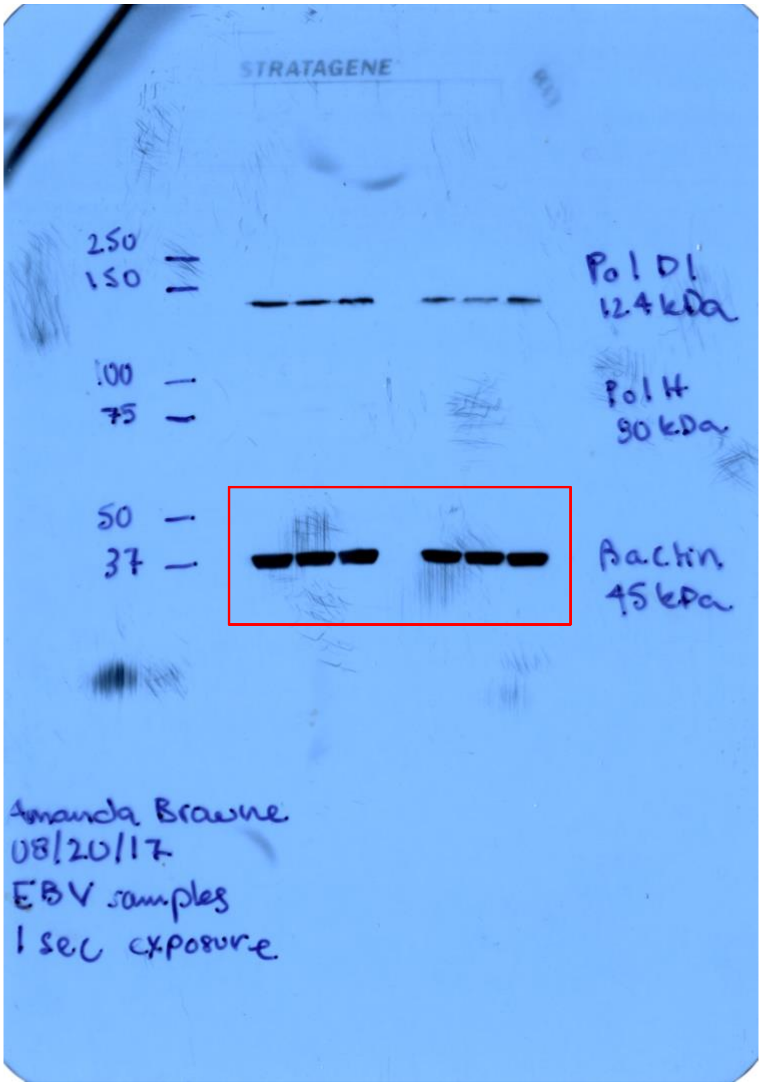

Demidova et. al, Supplementary Figure 6.

D **For Figure 2B: Polε**

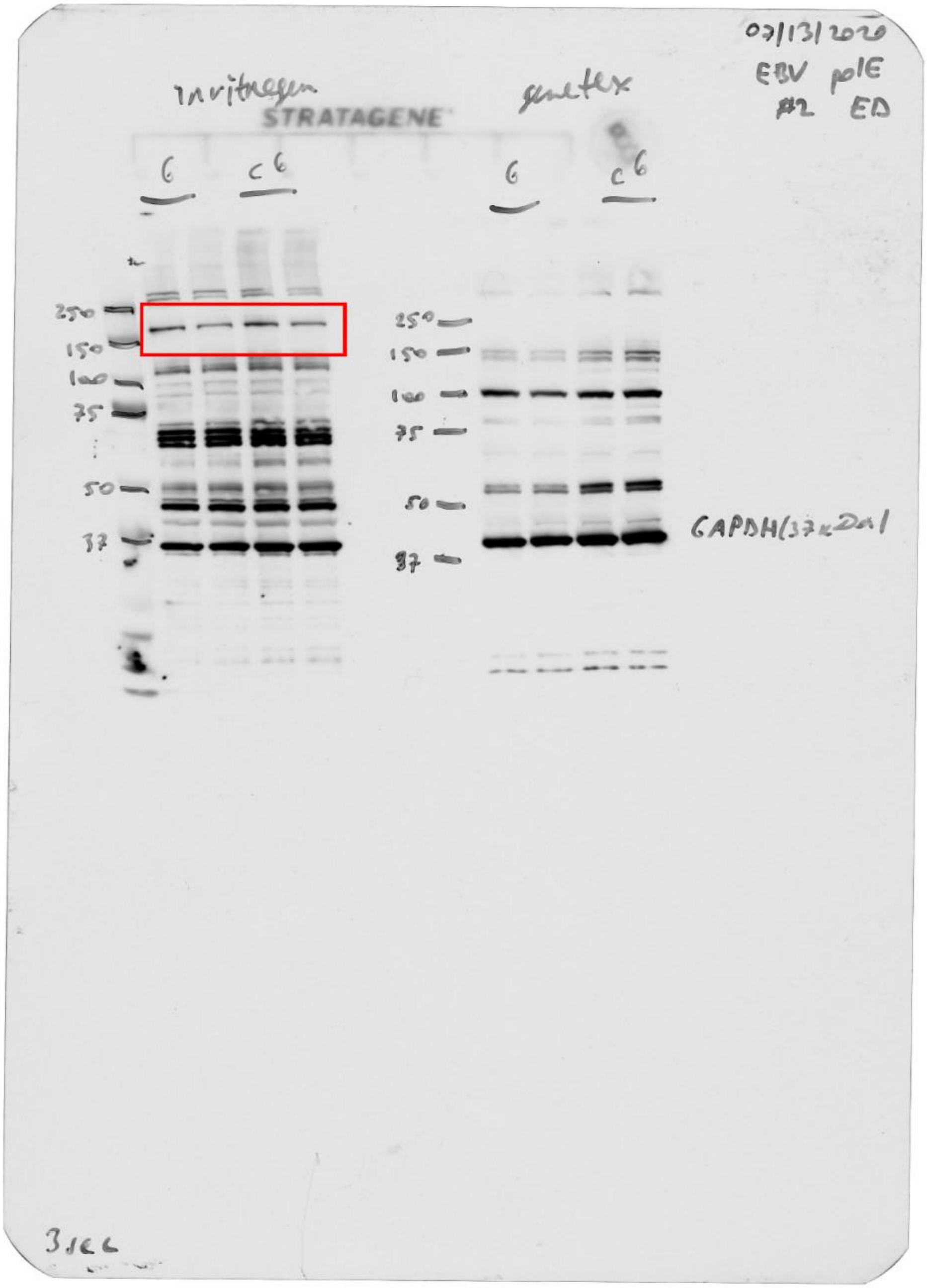

Raw image contains POLE cell line (G in the left) and control cell line (cG) on the right.

Demidova et al., Supplementary Figure 6

E **For Figure 2B: Polκ**

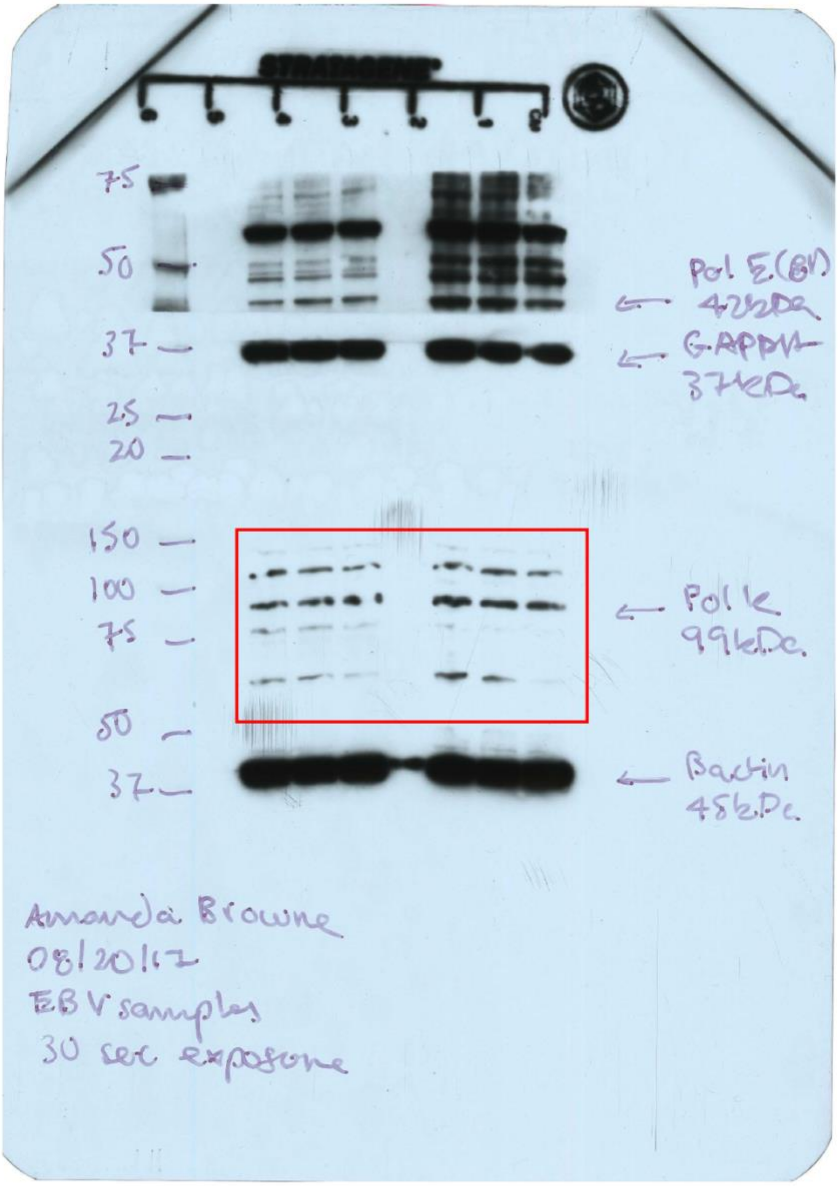

Demidova et al., Supplementary Figure 6

A For Figure 3A: Purification of Polδ

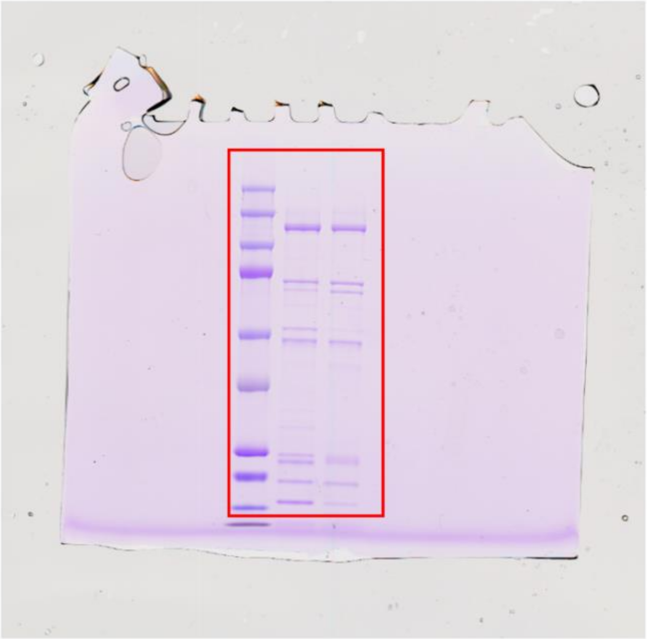

B For Figure 3B: Purification of Polη

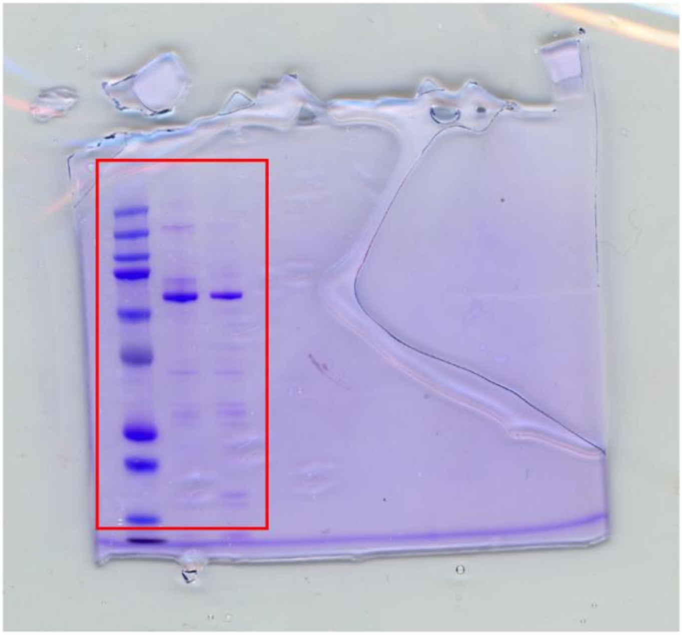

Demidova et al., Supplementary Figure 7

C For Figure 3A: Polδ primer extension assay (on right)

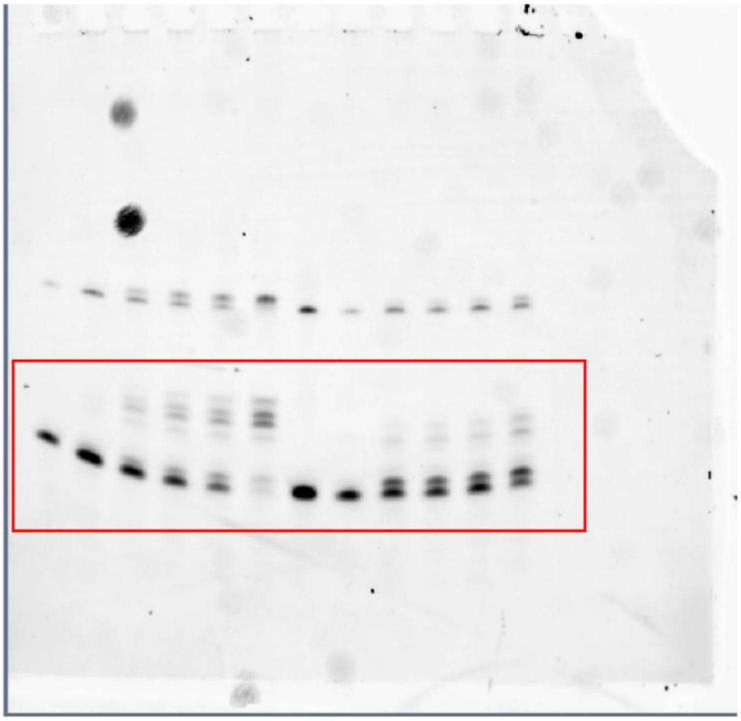

Demidova et al., Supplementary Figure 7

D **For Figure 3B on right- Pol η primer extension assay**

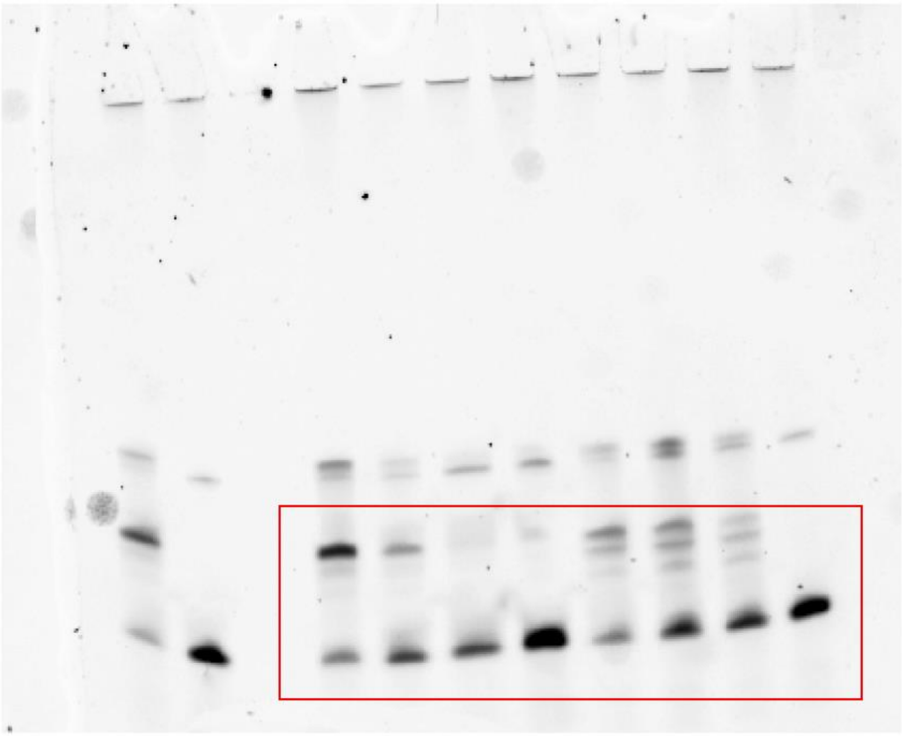

Raw image was flipped/cropped as appropriate for presentation in the final figure.

Demidova et al., Supplementary Figure 7

E **For Figure 3C: Polη lesion bypass assay.**

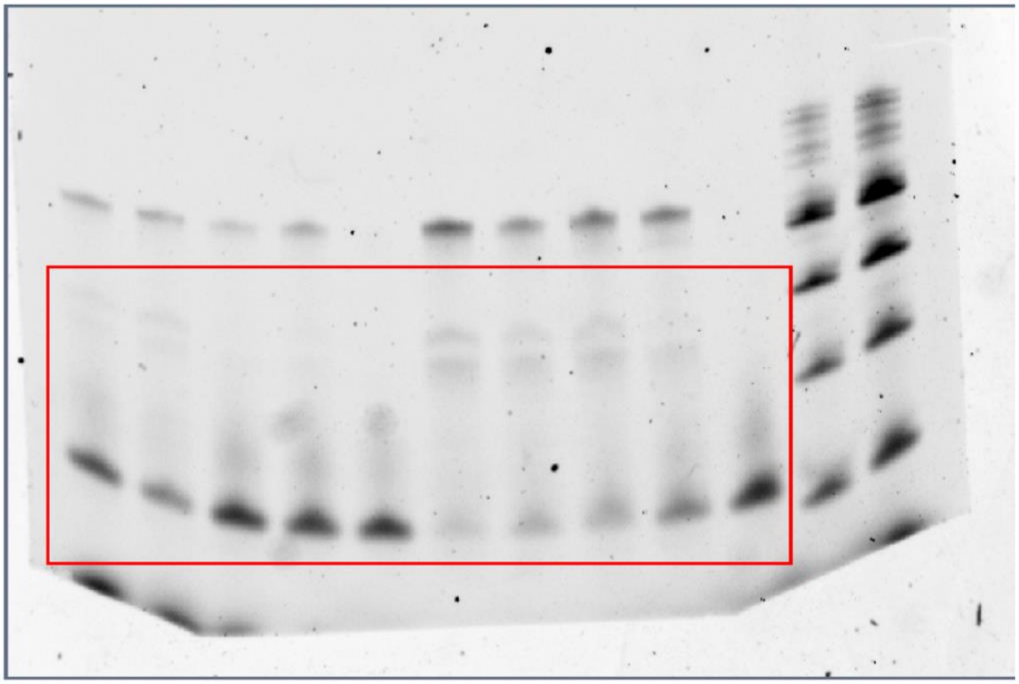

Raw image was flipped/cropped as appropriate for presentation in the paper.

Demidova et al., Supplementary Figure 7

F **For Supplementary Figure 5: Pol δ complex primer extension competition assay**

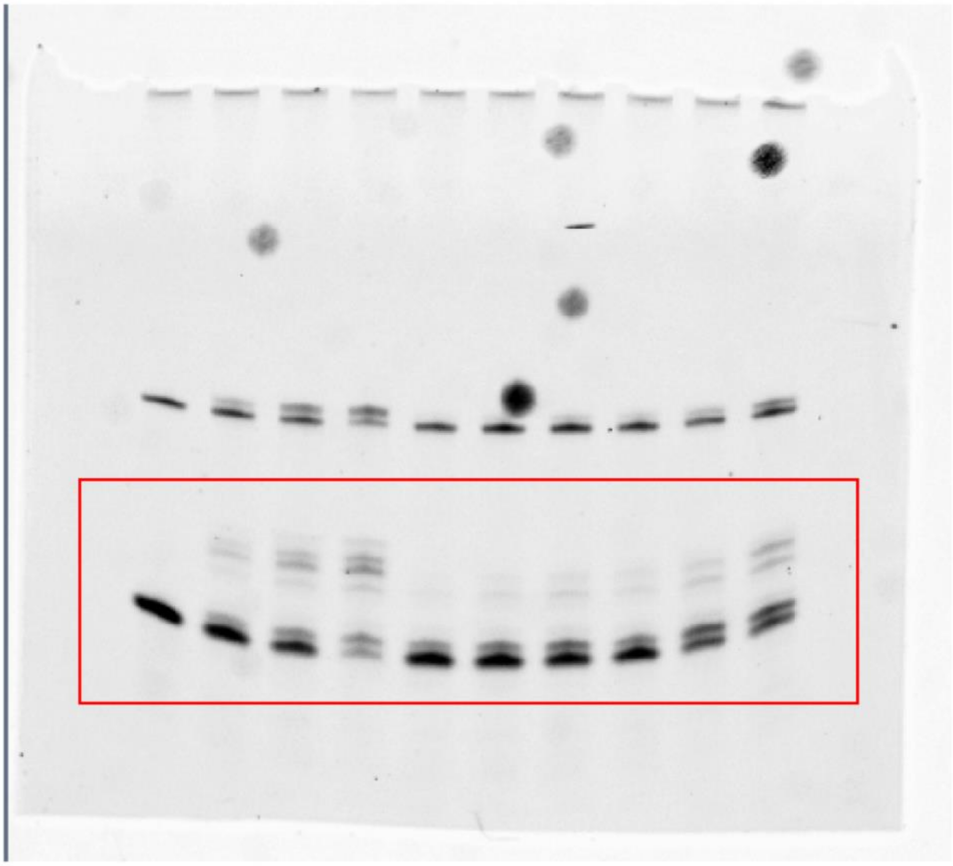

Demidova et al., Supplementary Figure 7

A

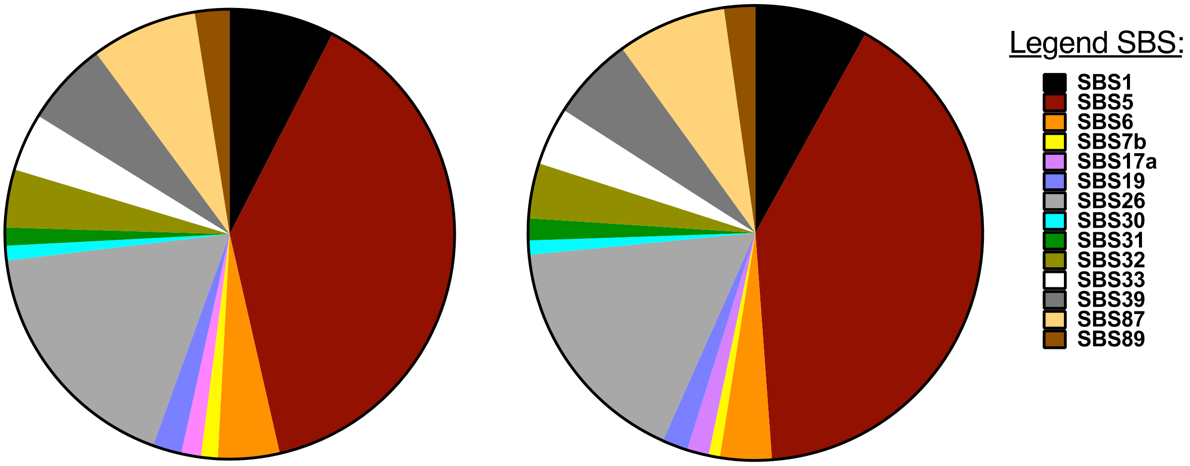

SBS signatures in Pt #1 tumor

SBS signatures in Pt #2 tumor

B

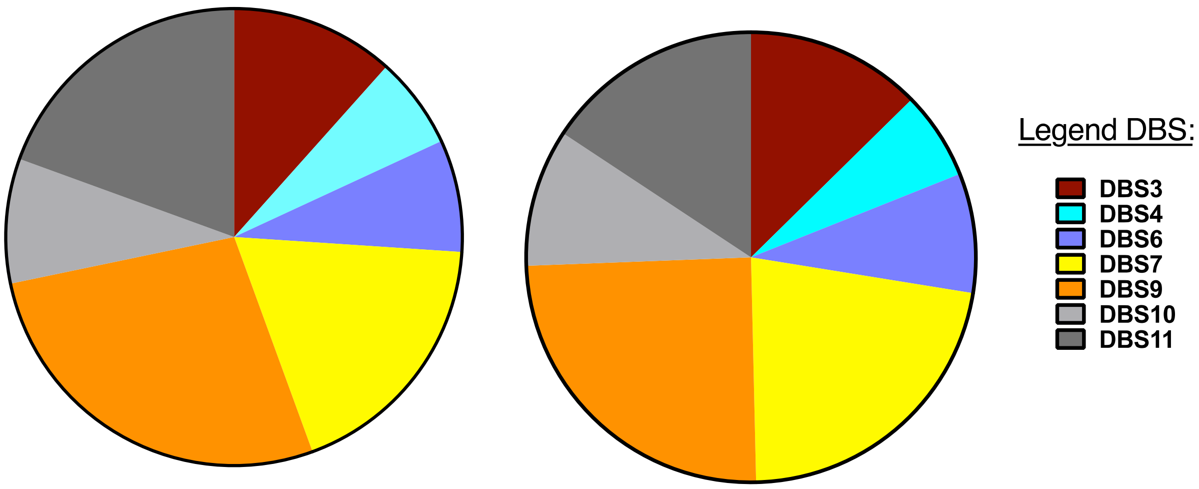

DBS signatures in Pt #1 tumor

DBS signatures in Pt #2 tumor

Demidova et. al, Supplementary Figure 8

Supplementary Table 3. Complete summary of results for the DNA polymerase variants identified in the eoRCC patients.

Gene	*POLD1*	*POLH*	*POLE*	*POLK*	ACMG criteria support
Variant	V759I missense	G209V missense	W1624X stopgain	E29K missense	PP2 or PM4 for *POLE*
Frequency in public databases	very rare	very rare	very rare	very rare	PM2
Computational predictions	damaging	damaging	damaging	damaging	PP3
γH2AX in patient primary PBMCs	increased		increased	increased	PM1
γH2AX on knockdown in Caki cells	decreased	decreased	decreased	decreased	PM1
Protein expression in EBV-cell lines	no difference	decreased	no difference	no difference	PM1
Cell counts in EBV-cell lines	no difference		no difference	no difference	N/A
CTB viability in EBV-cell lines	increased viability		increased viability	increased viability	PM1
DNA fiber assay in EBV-cell lines	slower replication speed, slower fork restoration		slower replication speed, slower fork restoration	no difference in replication	PM1
Cell cycle in EBV-cell lines	no difference		no difference	no difference	PM1
Tumor sequencing (LOH)	no LOH	no LOH	no LOH	no LOH	N/A
TMB analysis	12.85 mut/Mb		14.44 mut/Mb	-	PM1
Structural modeling	may disrupt D757 residue that coordinates Mg^{2+} ions in the active center of the polymerase domain	may alter stability of the α-helix in the polymerase active center	N/A (stop gain variant)	-	N/A
Biochemical assays	less robust polymerase activity, impaired function versus wild type complex	slower lesion bypass, suggestive of better processivity over wild type	Same as above	see below	PM1
Biochemical assays previously reported	-	-	-	reduced catalytic efficiency and reduced replication	PM1

				fidelity versus wild type (20)	

ACMG - American College of Medical Genetics, CTB – cell titer blue; DDR – DNA damage and repair; eoRCC – early-onset renal cell carcinoma; LOH – loss of heterozygosity; NA – non-applicable; NS – non-significant; PBMCs – peripheral blood monocytes; TMB – tumor mutation burden

ACMG criteria codes: PM1 - moderate evidence of pathogenicity (located in a mutational hot spot and/or critical and well-established functional domain (*e.g*. active site of an enzyme) without benign variation); PM2 - moderate evidence of pathogenicity (absent from controls (or at extremely low frequency if recessive) in Exome Sequencing Project, 1000 Genomes or ExAC); PM4 - moderate evidence of pathogenicity (protein length changes due to in-frame deletions/insertions in a non-repeat region or stop-loss variants); PP2 - supporting evidence of pathogenicity (missense variant in a gene that has a low rate of benign missense variation and where missense variants are a common mechanism of disease); PP3 - supporting evidence of pathogenicity (multiple lines of computational evidence support a deleterious effect on the gene or gene product (conservation, evolutionary, splicing impact, etc)).

Supplementary Table 4. Protein stability or ddG values for the PolD1 V759I and Pol η G209V variant proteins that were assessed biochemically in the study.

Protein and Variant	ddG
PolD1 V759I	-1.363
Pol η G209V	5.667

In legend:			
Variants assosiated with >10 mut/Mb			
ddG	conformation destabilizing		
ddG	conformation stabilizing		

Supplementary table 5. *POLE and POLD1 variants in hypermutated ccRCC in TCGA.*

PolD1 Amino acid change	Protein domain	GnomAD allele count	Associated TMB (from PMID: 29056344)	ddG
R6W	no specific domain	4	48.6	0.211
V13L	no specific domain	1	25.2	0.900
R78H	no specific domain	6	10.8	2.431
G129A	no specific domain	4	55.9	1.464
P151S	exonuclease	1	458.6	4.126
R166W	exonuclease	10	20.7	-1.625
W304M	exonuclease	0	33.3	2.645
W304S	exonuclease	0	27	6.556
H565L	polymerase	0	73	-2.635
S665N	polymerase	1	23.4	0.555
A675S	polymerase	0	17.1	2.459
A810S	polymerase	2	13.5	3.906
R823G	polymerase	0	229.7	2.057
R823L	polymerase	0	18.9	0.645
R875C	polymerase	1	541.36	0.307
D893N	polymerase	3	82.9	-1.223
R978C	polymerase	3	94.6	1.991
R1008H	no specific domain	0	21.6	1.885
A1046V	no specific domain	1	450.5	0.545

Pol ε Amino acid change	Protein domain	GnomAD allele count	Associated TMB (from PMID: 29056344)	ddG
D45N	N-terminal domain (NTD)	0	99.1	-1.740
M66R	N-terminal domain (NTD)	1	10.8	5.387
P557S	polymerase - palm	1	81.1	2.421
H580R	polymerase - palm	0	31.5	0.991
P696R	polymerase - palm	0	11.7	1.906
F753L	polymerase - palm	0	92.8	0.019
S803L	polymerase - fingers	0	245.9	-6.842
I843V	polymerase - palm	6	27	1.245
F1109Y	polymerase - thumb	0	119.8	-0.791
D1165N	polymerase - thumb	3	118.9	-1.351
E1173G	polymerase - thumb	1	11.7	-0.256
R1364C	C-terminal domain (CTD)	7	60.4	-1.784
T1429S	C-terminal domain (CTD)	2	26.1	2.829
R1556W	C-terminal domain (CTD)	1	337.8	-0.483
R1626C	C-terminal domain (CTD)	3	126.1	-2.306
R1691H	C-terminal domain (CTD)	1	49.5	0.652
R1691L	C-terminal domain (CTD)	0	18.9	-3.010
R1870H	C-terminal domain (CTD)	4	34.2	3.604
N1921K	C-terminal domain (CTD)	0	170.3	3.033
P2088L	C-terminal domain (CTD)	1	74.8	-0.506
D2128N	C-terminal domain (CTD)	0	45.9	-1.626
E2200K	C-terminal domain (CTD)	2	15.3	-2.542
G2240R	C-terminal domain (CTD)	1	25.2	4.808

In legend:	
Variants assosiated with >10 mut/Mb	
ddG	conformation destabilizing
ddG	conformation stabilizing

Supplementary table 6. DNA-substrates used in biochemical assays.

Duplex name	Oligonucleotide name	Sequence	Source
SA#1	Cy3-SA-pold-elong	5’-*ATTATGCGGCCGTGTACAACGGGTGTCGGGGC-3’	PMID: 27591252
	SA-pold-elong	5’-GCTCTGATGCCGCATAGTTAAGCCAGCCCCGACACCCG-3’	
	CY5-SA-polh	5’-*CAGCACCACAAACCATACAAAAACA-3’	PMID: 19966286
SA#2	SA-polh	5’-GTGGTGGTGTTTGGTATGTTTTTGTCATATAATCTTTGGGCTATTACCG-3’	
SA#3 and SA#4	CY5-8oxG-SA-polh	5’-*GCAGGTCGACTCCAAAG-3’	PMID: 23913529
SA#3	G-SA-polh	5’-CGTCCAGCTGAGGTTTCCGATTGGGCCATGGCT-3’	
SA#4	8oxG-SA-polh	5’-CGTCCAGCTGAGGTTTCC-8oxoG-ATTGGGCCATGGCT-3’	

Supplemental Table 7. ddG application file.

-in:file:s /home/andrakem/Documents/clients/Arora/ddgPolE/min_cst_0.5.AF2hPolE_0001.pdb # the PDB file of the structure on which point mutations should be made

-resfile /home/andrakem/Documents/clients/Arora/ddgPolE/mutfilePolE # the list of point mutations to consider in this run

-ddg:weight_file soft_rep_design # Use soft-repulsive weights for the initial sidechain optimization stage

-database /home/dunbracklab/Rosetta/main/database #the full path to the database is required

-ddg::iterations 25 # 50 is the recommended number of iterations

-ddg::dump_pdbs true # write out PDB files for the structures, one for the wildtype and one for the pointmutant for each iteration

-ddg::local_opt_only false # recommended: local optimization restricts the sidechain optimization to only the 8 A neighborhood of the mutation (equivalent to row 13)

-ddg::min_cst false # use distance restraints (aka constraints) during the backbone minimization phase

-constraints::cst_file /home/andrakem/Documents/clients/Arora/ddgPolE/input.cst # the set of constraints to use during minimization which should reflect distances in the original (non-pre-relaxed) structure

-in::file::fullatom # read the input PDB file as a fullatom structure

-ddg::mean true # do not report the mean energy

-ddg::min true # report the minimum energy

-ddg::sc_min_only false # do not minimize only the backbone during the backbone minimization phase

-ddg::ramp_repulsive true # perform three rounds of minimization (and not just the default 1 round) where the weight on the repulsive term is increased from 10% to 33% to 100%
